# Strategic decoupling between grant and publication language in AI and cancer research: a cross-national LLM-assisted analysis

**DOI:** 10.3389/frma.2026.1893522

**Published:** 2026-07-16

**Authors:** Xinghang Wang, Yu Sun

**Affiliations:** State Key Laboratory of Swine and Poultry Breeding Industry, and Guangdong Provincial Key Laboratory for the Development Biology and Environmental Adaptation of Agricultural Organisms, College of Life Sciences, South China Agricultural University, Guangzhou, Guangdong, China

**Keywords:** artificial intelligence, cancer immunology, funding rhetoric, grant writing, large language models, research evaluation, research integrity, scientific communication

## Abstract

Funding systems increasingly reward scientists who frame technical questions as narratives of national priority and societal impact, yet whether this policy-facing language carries through to how science is actually reported, or merely serves as a temporary rhetorical layer for grant competition, remains unclear. Using Google Gemini as a large language model-assisted text-analytic tool, we scored 400 matched pairs of funded grant abstracts and their linked peer-reviewed publications from China and the United States, across two contrasting fields: artificial intelligence and cancer immunology. Each text was scored 1–10 for macro-narrative (“hype”) language and for mechanistic scientific logic, and a blinded 20% subset rated by two human raters showed broad agreement with the model-based scores. Funding language differed more by field than by country: artificial intelligence grants in both nations showed substantially higher policy-facing rhetoric than cancer immunology grants, which remained anchored in mechanistic logic. This rhetorical inflation, however, largely disappeared at the publication stage, where macro-narrative scores dropped sharply across both countries and disciplines—a grant-to-publication shift we term strategic textual decoupling. These findings suggest that funding systems may encourage rhetorical compliance without necessarily improving the scientific record, and that the gap between the language required to win funding and the language used to communicate science represents a hidden cognitive tax. Reducing unnecessary narrative inflation in grant evaluation, and tailoring assessment to disciplinary realities, could improve research integrity and the efficient use of researchers' time.

## Introduction

The tension between top-down, mission-driven funding priorities and bottom-up, curiosity-driven scientific discovery is a fundamental challenge in global health and science policy (Fortunato et al., 2018). Securing research funding has become fiercely competitive. To stand out, scientists and medical researchers are often encouraged—or explicitly required—by funding agencies to frame their proposals using positive, hyperbolic language and grand narratives that align with macroeconomic strategies (Vinkers et al., 2015).

As the global buzz around disruptive technologies intensifies, there is growing concern among the medical and scientific community that administrative pressures and “hype” may erode the mechanistic rigor of basic research (Lipton and Steinhardt, 2019). However, it remains unclear how this hyperbolic language in the biomedical field compares to rapidly evolving, “hot” technological areas such as artificial intelligence (AI). Does the pattern differ by subject area, or do all fields suffer the same administrative pressure? Furthermore, is this linguistic inflation a localized bureaucratic pathology, or a universal symptom of modern science funding?.

### Quantifying the language of funding

To explore these questions, we conducted a bibliometric analysis driven by a Large Language Model (LLM) (Korinek, 2023). We curated a dataset comprising 400 matched pairs of funded grant abstracts and their subsequent high-impact publications. We purposefully selected two contrasting fields: AI, representing a highly politicized technological frontier, and Cancer Immunology, representing a deeply grounded medical basic science.

For the United States, we retrieved R01 grant abstracts from the NIH RePORTER (Cancer) and the NSF Award Search (AI) initiated between 2018 and 2024. For China, we collected corresponding General and Key Program abstracts from the National Natural Science Foundation of China (NSFC). We then matched each grant to a linked, peer-reviewed English publication in the Web of Science Core Collection. Field membership was defined by the funding programme of origin rather than by topic inference. Grants at the interface of the two areas (e.g., machine-learning methods applied to oncology) were identified and excluded, leaving two mutually exclusive fields. For grants linked to more than one eligible publication, we selected the highest-cited paper to provide a single, standardized downstream text comparable across grants; this prioritizes consistency of the comparison anchor over representativeness of grant output. Using Google's LLM (Gemini 3.1 Flash), we blindly scored the texts from 1 to 10 across two dimensions: the density of macroscopic “bureaucratic/hype” narratives vs. the presence of microscopic, mechanistic scientific logic. The complete prompts provided to the model, including the full scoring rubric and operational definitions of the macro-narrative and mechanistic-logic dimensions, are reported verbatim in Supplementary material S1 and in the project repository, to enable full reproduction of the scoring procedure.

As a face-validity check, two blinded human raters independently scored a random 20% subset of matched grant–publication pairs using the same 1–10 rubric. Agreement was assessed by comparing LLM scores with the mean human score using intraclass correlation coefficients for absolute agreement, supplemented by the mean absolute error and the proportion of scores within one point. The human validation subset showed broad agreement with the LLM-based scoring. The intraclass correlation coefficients for absolute agreement were 0.83 for macro-narrative scores and 0.79 for mechanistic-logic scores; the mean absolute errors were 0.57 and 0.65 points, respectively; and 87 and 83% of LLM scores, respectively, were within one point of the mean human score. Inter-rater agreement between the two human raters, assessed on the same subset using a two-way random-effects intraclass correlation coefficient for absolute agreement, was 0.84 for macro-narrative and 0.80 for mechanistic-logic scores; LLM–human agreement was therefore comparable to human–human agreement. These results support the use of LLM scores as an exploratory indicator of rhetorical positioning rather than as a definitive measurement instrument. Grant-to-publication differences were tested with paired Wilcoxon signed-rank tests, stratified by field and country; between-group differences used Mann–Whitney *U*-tests. All tests were two-sided, with *p*-values Benjamini–Hochberg adjusted. Effect sizes are reported as rank-biserial correlation (*r*) for paired comparisons.

### Funding narratives differed more by field than by country

**Figure 1:**
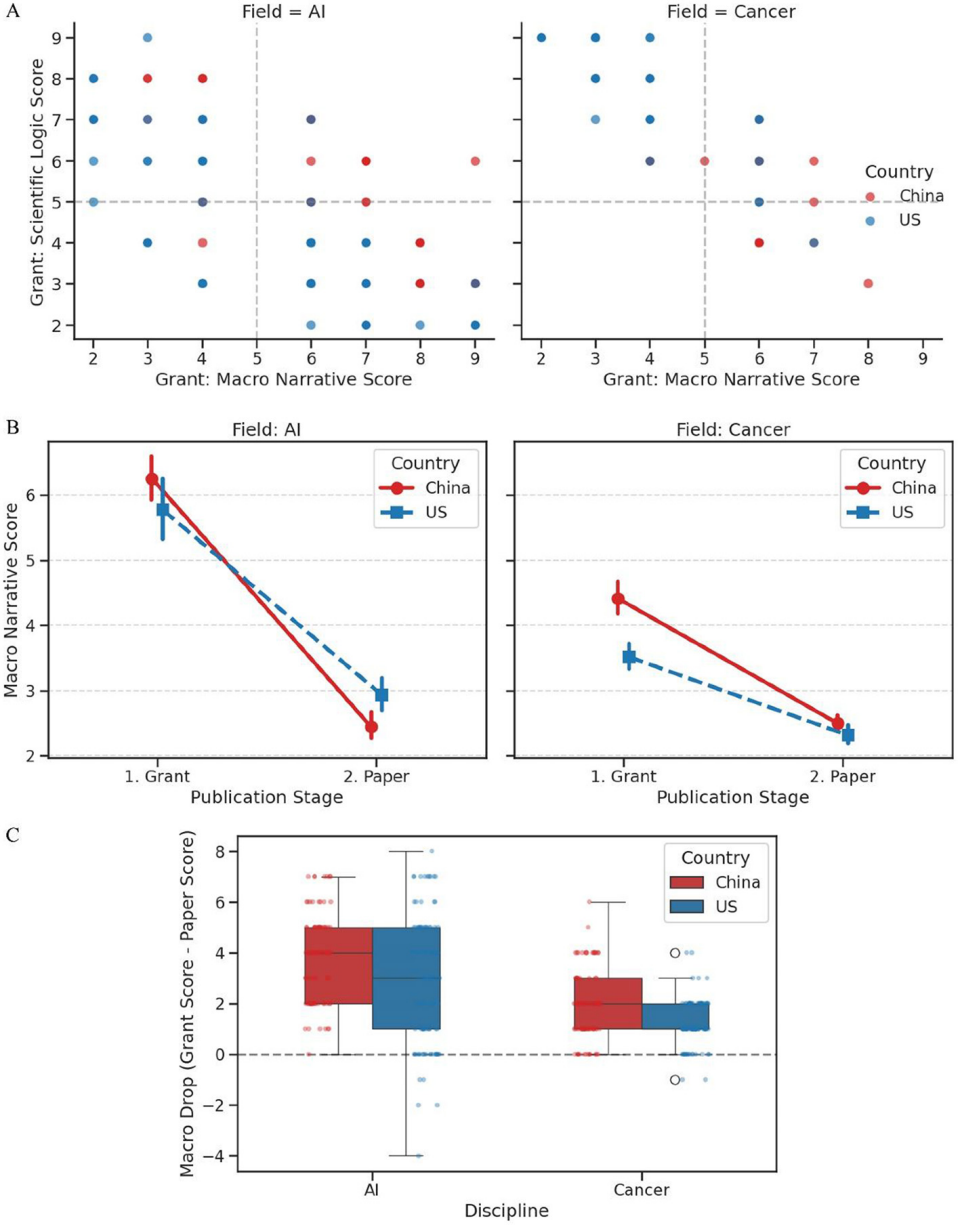
The linguistic profiles and strategic textual decoupling in funding narratives. **(A)** Quadrant scatter plots illustrating the initial linguistic positioning of funded grant abstracts. In Cancer Biology, Chinese grants (red) exhibit a distinctly higher density of macro-narrative positioning compared to US grants (blue), though both maintain robust scientific logic. The hype-driven AI field pushes both nations toward much higher macro-narrative positioning. Dashed lines denote the median baseline score of 5. **(B)** The linguistic trajectory demonstrating the shift in Macro-Narrative scores from funding proposals (Stage 1) to their corresponding high-impact publications (Stage 2). Chinese cancer researchers exhibit a sharp, statistically significant drop in bureaucratic language upon publication (Benjamini–Hochberg adjusted *p* < 0.01). Points represent the mean scores, and error bars indicate the 95% confidence intervals. **(C)** The Decoupling Index (macro drop) comparing linguistic friction across disciplines. Chinese scientists face a significantly higher decoupling burden than US scientists in basic sciences (Benjamini–Hochberg adjusted *p* < 0.01). Box plots show the median, interquartile range (IQR), and 1.5 × IQR, with overlaid dots representing individual grant-publication pairs.

Our initial assumption was that the divergence in grant language would be strongly country-specific, reflecting different administrative cultures. Surprisingly, the data reveals a compelling subject-specific pattern: the discipline itself is the primary driver of funding hype (Figure 1A).

In basic medical sciences like cancer biology, both US and Chinese scientists maintain a relatively grounded, science-driven profile in their grant applications. While Chinese NSFC cancer grants exhibit a measurably higher density of systemic terminology to align with national health goals, the overall linguistic ecosystem remains robustly anchored in mechanistic biology. Standard public health terms such as “unmet clinical need” or “health impact” are utilized appropriately to justify research relevance without overwhelming the scientific core.

In stark contrast, the AI field demonstrates a higher prevalence of broad, vision-oriented narratives. Regardless of whether the funding agency is the US NSF or the Chinese NSFC, AI grant abstracts are saturated with visionary hype and macroscopic promises. Instead of merely proposing algorithmic optimizations, researchers frequently frame their grants with administrative or visionary buzzwords. In the US context, grants are framed to “kindle and fuel a transformation” and address “nationally-important” issues, utilizing concepts like “empower.” In parallel, Chinese researchers frequently rely on corresponding terms such as “industrial empowerment” (产业赋能), “chokepoint challenges” (瓶颈难题), “major national needs” (国家重大需求), and “leapfrog development” (跨越式发展). This pattern indicates that when a scientific discipline becomes heavily entangled with geopolitical and economic expectations, the language of its researchers shifts from technical description toward policy-facing justification Wang and Barabási (2021).

### The phenomenon of strategic linguistic decoupling

If grant applications in hype-driven fields are dominated by macroscopic narratives, does this mean the actual science being conducted has lost its rigor? Our analysis provides a more reassuring pattern through the discovery of a phenomenon we term strategic textual decoupling.

When tracing the linguistic trajectory from the initial grant proposal to the final high-impact publication, we observe a drastic shift (Figure 1B). Despite the heavy reliance on grand narratives to secure funding—especially in the AI sector—scientists from both nations experience a cliff-like drop in macroscopic language upon publication, coupled with a surge in technical scientific rigor.

This decoupling shift is highly significant across disciplines (Benjamini-Hochberg adjusted *p* < 0.01), exposing the “dual-track” behavior of modern researchers. The grant-to-publication decline in macro-narrative score corresponded to a large effect in AI (*r* = 0.78) and a moderate effect in cancer immunology (*r* = 0.56). Peer review serves as a equalizing force, pulling researchers back to medical reality (Tennant et al., 2017). The sharp decline in hype from grant to paper indicates that the administrative demand for grand narratives does not dictate the actual execution of bottom-up science. Instead, top-tier scientists have evolved a sophisticated strategy: they adopt compliance language to navigate the bureaucratic maze and secure resources, while steadfastly shielding their actual laboratory work to maintain global scientific integrity.

### The cognitive tax on researchers

While this strategic decoupling highlights the remarkable resilience of the scientific community, it also exposes a possible systemic inefficiency. The gap between what scientists must write to obtain funding and what they write to advance science represents a hidden “cognitive tax” (von Hippel and von Hippel, 2015), which we quantified by the decoupling index across disciplines (Figure 1C).

Our findings suggest that the increasingly complex requirements for policy alignment and visionary promises in funding applications force researchers expend time and attention on linguistic packaging rather than scientific ideation. This friction is particularly acute in China, where the administrative coupling of basic research to national strategic objectives requires scientists to heavily dress micro-level biological or computational inquiries in macro-political frameworks. However, as our data shows, this rhetorical inflation ultimately vanishes at the publication stage, rendering the initial bureaucratic packaging functionally redundant in the scientific record.

### Implications for grant evaluation

Our findings are consistent with recent initiatives, including the State Council of China's guidelines, aimed at reducing administrative burden in research. The decline in macro-narrative language from grant to publication indicates that the policy-facing rhetoric required at the funding stage leaves little trace in the eventual scientific record. This pattern is compatible with the interpretation that narrative requirements in grant evaluation impose costs on researchers' time without a corresponding gain in the scientific output, although our descriptive design cannot establish this causally. Approaches such as simplifying application formats, reducing non-scientific justification requirements, and tailoring evaluation criteria to disciplinary norms may help align the language of funding more closely with the logic of the underlying research.

### Limitations

The study has several limitations. First, selecting the highest-cited linked publication introduces survivorship bias: grants that produced no indexed English-language publication, or only lower-impact work, are not represented. Our results therefore describe the grant-to-publication rhetorical trajectory among grants that culminate in high-impact papers and should not be generalized to all funded projects. Second, because publications typically lag funding by 2 to 5 years, grants funded toward the end of our 2018–2024 window are under-represented among matched pairs; the matched sample skews toward earlier grants with established publication records, and the most recent funding cohorts are only partially captured. Third, although we expanded human validation to 20% of pairs, this remains a face-validity check rather than a full psychometric validation. The LLM-derived scores should be read as an exploratory indicator of rhetorical positioning rather than a definitive measurement, and larger-scale, multi-rater validation remains an important direction for future work.

## Conclusions

Our analysis suggests that funding and publication language are shaped by different incentive systems. In this matched study, field differences were more pronounced than country difference. Yet this rhetorical inflation largely disappeared in subsequent peer-reviewed publications, where technical and mechanistic language became more prominent. We describe this pattern as strategic textual decoupling. It may reflect a hidden cognitive and administrative tax: researchers learn to satisfy the narrative expectations of funding systems while preserving scientific rigor in the published record. For research integrity and peer review, this matters because the incentives that shape funding applications also shape the conditions under which science is produced. Funding agencies and reviewers should reduce unnecessary narrative inflation and evaluate scientific merit in ways that are more transparent, discipline-sensitive, and closely aligned with the logic of the research itself.

## Data Availability

The datasets presented in this study can be found in online repositories. The names of the repository/repositories and accession number(s) can be found below: https://github.com/sunyucourse/Funding-Analysis.
