# Supplementary material for: Strategic decoupling between grant and publication language in AI and cancer research: a cross-national LLM-assisted analysis
Source: Front Res Metr Anal. 2026 Jul 16;11:1893522. doi: 10.3389/frma.2026.1893522 (PMC13422417; doi:10.3389/frma.2026.1893522)
Supplement: Supplementary file 1 [file Data_Sheet_1.docx]

**Supplementary Material S1**

**Large language model scoring: prompts, dimension definitions, and procedure**

*Manuscript: “Strategic decoupling between grant and publication language in AI and cancer research”*

This document reports, verbatim, the prompt used to score every text in the study, together with the operational definitions of the two scoring dimensions, the output format, and the processing procedure, so that the scoring can be reproduced independently.

# 1. Model and generation settings

| **Model** | Google Gemini 3.1 Flash |
| --- | --- |
| **Access / interface** | API endpoint |
| **Temperature** | 0 (deterministic scoring) |
| **Call structure** | Each text scored in a single, independent request: the system prompt below plus one grant abstract OR one publication abstract as the user message. Grant and publication texts were scored separately and never combined in one call. |

# 2. System prompt (verbatim, as used)

Scoring was performed with a Chinese-language system prompt because the corpus contained both Chinese (NSFC) and English (NIH, NSF, and all publications) texts; the prompt instructs the model to score the underlying narrative motivation and scientific logic irrespective of surface language. The original prompt is reproduced first, followed by an English translation.

## 2.1 Original prompt (Chinese, as used)

你是一位深谙全球科技政策与科学计量学（Science of Science）的客观评估专家。你的任务是对输入的科研文本进行双维度的量化打分。无论文本是中文还是英文，请忽略语言差异，穿透其底层的“叙事动机”和“科学逻辑”。

请根据以下两个维度，分别给出 1-10分 的整数评分：

【维度一：宏大叙事与政策套话得分 (Macro_Score)】

高分 (8-10分)：文本充满自上而下的政策响应动机；大量使用缺乏微观实体的宏观热词（如：赋能、全链条、卡脆子、体系化、新变化新挑战、战略需求）；强调解决宏观产业或社会痛点，掩盖了具体科学机制细节。

低分 (1-3分)：文本纯粹聚焦学术，几乎没有政策性包装，开篇直奔具体的科学空白或技术矛盾。

【维度二：第一性原理与底层机制得分 (First_Principle_Score)】

高分 (8-10分)：文本逻辑自下而上；探讨极其具体的物理/生物/算法底层机制（回答 Why 和 How）；包含高密度的实体科学名词（如特定的蛋白质受体、数学方程参数、算法网络架构）；对研究边界有克制、清晰的界定。

低分 (1-3分)：文本停留在表象的功能描述（回答 What）；用模糊的形容词代替严密的逻辑推演；像在拼接技术模块，缺乏对底层规律的探索。

【输出要求】

必须严格输出纯 JSON 格式，不要有任何多余的解释、markdown符号或换行符，格式如下：

{"Macro_Score": 8, "First_Principle_Score": 3, "Reasoning": "打分的一句话简要理由"}

## 2.2 English translation (for reference)

You are an objective assessment expert with deep knowledge of global science-and-technology policy and the science of science. Your task is to assign quantitative scores to the input research text along two dimensions. Regardless of whether the text is in Chinese or English, ignore the surface language and look through to its underlying “narrative motivation” and “scientific logic.”

Assign an integer score from 1 to 10 for each of the two dimensions below:

[Dimension 1: Macro-narrative and policy-boilerplate score (Macro_Score)]

High (8-10): The text is saturated with top-down policy-response motivation; it makes heavy use of macro-level buzzwords lacking concrete micro-level entities (e.g., empowerment, whole-chain, chokepoint/bottleneck, systematization, new circumstances and new challenges, strategic demand); it emphasises solving macro industrial or societal pain points, obscuring specific scientific-mechanism detail.

Low (1-3): The text focuses purely on scholarship, with almost no policy packaging, opening directly with a specific scientific gap or technical contradiction.

[Dimension 2: First-principles and underlying-mechanism score (First_Principle_Score)]

High (8-10): The logic is bottom-up; it explores highly specific physical/biological/algorithmic underlying mechanisms (answering Why and How); it contains a high density of concrete scientific terms (e.g., specific protein receptors, equation parameters, network architectures); it delimits the research scope in a restrained, clear way.

Low (1-3): The text stays at the level of surface functional description (answering What); it substitutes vague adjectives for rigorous logical deduction; it reads like assembling technical modules, lacking exploration of underlying principles.

[Output requirements]

Output strictly in pure JSON, with no extra explanation, markdown, or line breaks, in this format:

{"Macro_Score": 8, "First_Principle_Score": 3, "Reasoning": "a one-sentence justification"}

# 3. User message

Each text was supplied to the model as the user message in the following form. The full, unedited abstract text was inserted in place of {TEXT}; no other instructions were added at the user-message level.

请评分以下文本 / Please score the following text:

{TEXT}

*[Confirm this matches the exact user-message wrapper used; if the abstract was passed with no preamble at all, state that instead.]*

# 4. Operational definitions of the two dimensions

Macro-narrative (“hype”) score — Macro_Score (1–10): the density of top-down, policy-facing, vision-oriented language and macro-level buzzwords relative to concrete scientific content. Higher scores indicate greater reliance on administrative or visionary framing.

Mechanistic-logic score — First_Principle_Score in the prompt (1–10): the density of bottom-up, mechanism-level scientific reasoning and concrete entities (specific methods, parameters, receptors, architectures), with clearly delimited scope. Higher scores indicate greater mechanistic rigour.

# 5. Output format and parsing

The model returned a single JSON object per text. The two integer fields were extracted for analysis; the free-text “Reasoning” field was retained for audit only and was not used in any quantitative analysis.

{"Macro_Score": <integer 1-10>, "First_Principle_Score": <integer 1-10>, "Reasoning": "<one sentence>"}

# 6. Worked examples (illustrative)

*The two rows below are synthetic, illustrative examples constructed only to show the expected input–output behaviour and JSON format. They are NOT drawn from the study dataset and contain no real scores from the analysis.*

| **Illustrative input (abstract excerpt)** | **Macro** | **Mech.** | **(Reasoning)** |
| --- | --- | --- | --- |
| “To serve the major national strategic demand for industrial empowerment and to overcome chokepoint bottlenecks, this project builds a whole-chain, systematized intelligent platform addressing new circumstances and new challenges …” (grant-style, vision-heavy) | 8 | 3 | *Heavy policy buzzwords; little concrete mechanism.* |
| “We show that PD-1 blockade restores CD8+ T-cell cytotoxicity by relieving SHP-2-mediated dephosphorylation; we quantify receptor occupancy as a function of antibody affinity (Kd) and derive the dose-response relationship …” (publication-style, mechanism-heavy) | 2 | 9 | *Specific receptors, parameters, and derivations; no policy framing.* |

*All prompts, scoring code, and aggregated per-pair scores are deposited in the project repository (see Data sharing statement).*
